# Predictors of Prolonged Grief in Suicide Loss Survivors: The Role of Social Invalidation, Meaning in Life and Time Since Loss

**DOI:** 10.1002/cpp.70137

**Published:** 2025-08-02

**Authors:** Rafael Salom, José Enrique Layrón, Ana Moreno Martínez, Robert A. Neimeyer, Sandra Pérez

**Affiliations:** ^1^ Clínica Nuestra Señora de la Paz, Orden Hospitalaria San Juan de Dios Madrid Spain; ^2^ Valencian International University (VIU) Valencia Spain; ^3^ Papageno Association Sevilla Spain; ^4^ Department of Psychology University of Memphis Memphis Tennessee USA; ^5^ Department of Personality, Assessment, and Psychological Treatments University of Valencia Valencia Spain

**Keywords:** meaning in life, prolonged grief, relationship closeness, social validation, Spanish Suicide loss survivors

## Abstract

This study explores predictors of prolonged grief symptoms in Spanish adults bereaved by suicide, focusing on demographic, circumstantial and psychosocial factors. Participants completed assessments on prolonged grief symptoms, depressive symptoms, posttraumatic growth, meaning in life, perceived social support, social (in)validation and time since loss. Results showed that social invalidation was the strongest predictor of prolonged grief symptoms. In contrast, a strong sense of meaning in life was linked to lower prolonged grief symptoms and depressive symptoms, underscoring its protective role. Time since loss also predicted prolonged grief symptoms, supporting the idea that grief lessens over time. Notably, posttraumatic growth correlated more with social validation than with time since the loss. Kinship differences were also significant: parents who lost a child reported the highest prolonged grief symptoms, followed by spouses, while those who lost more distant relatives showed lower grief intensity. These findings emphasize the psychological impact of social responses to suicide loss and the importance of meaning‐making in grief adaptation in Spanish suicide loss survivors.

## Summary

- Social invalidation was the strongest predictor of prolonged grief symptoms.
- Meaning in life was associated with lower grief and depressive symptoms.
- Time since loss predicted lower prolonged grief symptoms.
- Losing a child or partner was linked to higher emotional distress.
- Social validation was related to greater posttraumatic growth.

## 1 Introduction

Suicide is a global public health problem, with approximately 720,000 deaths per year (World Health Organization 2025). In Spain, 3.846 suicides were reported in 2024 (Instituto Nacional de Estadística 2025), making it the second leading cause of non‐illness‐related death. This has a serious impact on those close to them, known as ‘suicide loss survivors’ (Cerel et al. 2019; Levi‐Belz et al. 2021).

Although widely used, the term lacks a clear definition (Al‐Halabí and Fonseca‐Pedrero 2023). Some authors argue that self‐identifying as affected by the death, regardless of legal or biological ties, qualifies someone as a survivor, emphasizing psychological closeness over formal relationships (Andriessen 2009; Andriessen et al. 2017). Research debates whether suicide grief is unique or shares traits with other grief types. However, stigma, guilt, shame and perceived responsibility are more common in suicide loss, making it harder to manage (De Leo et al. 2013; Pitman et al. 2018; Sveen and Walby 2008). These factors can intensify prolonged grief and raise the risk of psychiatric conditions like depression or PTSD (Erlangsen et al. 2017).

Epidemiological data highlight the need to address suicide's impact through a ‘continuum of survivorship’, including (a) those exposed (e.g., acquaintances), (b) those psychologically affected regardless of relationship (e.g., witnesses, first responders), (c) short‐term bereaved with significant impact but resilience and (d) long‐term bereaved with chronic effects (Cerel et al. 2014). Each suicide affects around 135 people, 25 of whom may be deeply impacted (Brown et al. 2024; Cerel et al. 2019). Many survivors also face prolonged grief and increased suicidal ideation or attempts, indicating a bidirectional link between suicide grief and suicide risk (Pitman et al. 2014).

Among the factors that modulate the intensity of suicide grief, the nature of the relationship to the deceased has been highlighted as a critical determinant. Studies such as Brown et al. (2024) have shown that people who report a relationship with the deceased, whether familial or not, tend to experience more prolonged grief and higher depressive symptoms. This finding has led some authors to question the traditional classification of survivors based exclusively on family relationships, emphasizing the relevance of perceived psychological closeness rather than formal kinship (Bottomley and Neimeyer 2025; Brown et al. 2024; Neimeyer et al. 2017).

In addition, the time since the loss significantly influences the grieving experience among suicide loss survivors. While prolonged grief and depression generally decrease over time, the initial years are often the most distressing (Mitchell et al. 2004). Adaptation may depend on factors such as social support and the ability to find meaning in life after the loss (Whittaker et al. 2024). Suicide grief is also linked to a heightened risk of prolonged grief disorder, characterized by persistent emotional pain and difficulty accepting the death, which significantly impairs daily functioning (Entilli et al. 2023; Lee et al. 2017; Prigerson et al. 2009). This condition affects 10%–15% of the bereaved, with even higher rates following suicide (Mitchell et al. 2004). Additionally, comorbid disorders such as depression and posttraumatic stress disorder are frequently reported among survivors, at significantly higher rates than in other types of grief (Szuhany et al. 2021).

Social support is a key protective factor against prolonged grief. Survivors with strong formal and informal support networks report lower levels of prolonged grief and better adaptation to loss (Andriessen et al. 2019). Mutual support interventions and postvention services, support offered after a suicide, are essential in reducing stigma and fostering emotional expression in a safe setting, aiding recovery (Lee et al. 2017; Salom et al. 2024).

Post‐traumatic growth, the positive psychological change resulting from trauma, is increasingly studied in suicide grief. Despite its traumatic nature, some survivors develop resilience and find new meaning (Salom et al. 2024; Tedeschi and Calhoun 2004). Post‐traumatic growth is associated with social support, life purpose and meaning reconstruction (Neimeyer 2006; Neimeyer et al. 2023). Time since loss plays a role in processing grief and enabling growth, which often arises from prolonged, intentional reflection and life reevaluation (Hurst and Kannangara 2024). Although the relationships among meaning in life, post‐traumatic growth and prolonged grief are complex, evidence suggests that meaning reconstruction promotes post‐traumatic growth and may reduce prolonged grief (Bogensperger and Lueger‐Schuster 2014; Sawyer and Brewster 2019).

To date, only one study in Spain has focused on individuals bereaved by suicide (Salom et al. 2024). Despite increasing international evidence on the psychological consequences of suicide loss, there is a notable absence of research examining the interplay between prolonged grief and related emotional, cognitive and social variables in the Spanish population. Given the cultural relevance of social ties and interpersonal relationships in Spain, it is essential to assess whether previously established associations, such as those between prolonged grief symptoms, depressive symptoms, post‐traumatic growth, meaning in life, social support and social (in)validation, also emerge in this sociocultural context. Furthermore, although the literature has consistently identified robust associations between these variables, their joint contribution to prolonged grief symptoms has not been tested in Spanish suicide loss survivors. This study, therefore, aims to fill that gap by (1) examining the relationships between prolonged grief symptoms, depressive symptoms, PTG, meaning in life, social support, social validation/invalidation and time since the death; (2) identifying key predictors of prolonged grief symptoms; and (3) comparing levels of post‐traumatic growth, depression, meaning and social experiences across groups defined by time elapsed since the loss.

## 2 Method

### 2.1 Participants

The study included Spanish‐speaking adults who had lost a loved one to suicide. Inclusion criteria were as follows: (1) being an adult Spanish speaker or understanding Spanish and (2) having experienced suicide loss. Participants with diagnoses of major depressive disorder, PTSD or psychotic disorders were informed about the potential emotional impact of the questions. Participation was voluntary.

Of the 596 respondents, 289 were excluded due to incomplete responses, resulting in a final sample of 307 participants, mostly women (86%), aged 18–73 (*M* = 42.92, *SD* = 12.91). Sociodemographic data are shown in Table 1.

**TABLE 1:** Sociodemographic data.

	*n*	%
Relationship to the deceased		
Parent	90	38.3
Son/Daughter	27	8.8
Spouse/Partner	44	14.3
Sibling	64	20.8
Friend	49	15.9
Grandparent	8	2.6
Uncle/Aunt	14	4.6
Cousin	7	2.3
Nephew/Niece	2	0.7
Brother‐/Sister‐in‐law	2	0.7
Marital status		
Single	70	49.8
Married/Cohabiting	237	22.8
Educational level		
University degree	166	54.1
High school/Vocational training	103	33.6
Primary/Secondary education	38	12.3

All participants provided written informed consent to participate in the study. The study was approved by the University's Ethics Committee (code UCV/2020‐2021/010).

### 2.2 Materials

#### 2.2.1 Sociodemographic and Loss‐Related Interview

This is an *ad hoc* questionnaire designed for present research, which collects sociodemographic and clinical data, as well as information on the participant's relationship with the deceased, the time elapsed since the loss and the age of the deceased.

#### 2.2.2 The Patient Health Questionnaire (PHQ‐9)

The PHQ‐9 (Kroenke et al. 2001; Martinez et al. 2023) includes nine items rated on a 0–3 Likert scale, assessing depressive symptoms. Total scores range from 0 to 27. The Spanish version shows a Cronbach's alpha between 0.78 and 0.90.

#### 2.2.3 Post‐Traumatic Growth Inventory (PTGI)

The PTGI (Tedeschi and Calhoun 1996; Castro et al. 2015) is a 21‐item scale assessing positive personal changes after trauma, rated on a 0–5 Likert scale. The Spanish version showed strong internal consistency α = 0.95.

#### 2.2.4 Inventory of Social Support (ISS)

The ISS (Hogan and Schmidt 2016) is a five‐item scale assessing perceived support during grief on a 5‐point Likert scale, with higher scores indicating greater social support. The internal consistency is acceptable α = 0.77.

#### 2.2.5 Purpose‐In‐Life Test‐10 (PIL‐10)

The PIL‐10 (García‐Alandete et al. 2013) is a 10‐item adaptation of the original 20‐item PIL scale, assessing meaning and purpose in life. Items are rated on a 7‐point Likert scale, yielding total scores from 10 to 70. The Spanish version shows good reliability α = 0.85.

#### 2.2.6 The Social Meaning in Life Events Scale (SMILES)

The SMILES (Bellet et al. 2018; Pérez et al. 2025) includes 24 items assessing perceived social validation or invalidation when making sense of a significant loss or trauma. Responses are rated on a 5‐point Likert scale. The Social Invalidation subscale reflects feelings of judgement, while the Social Validation subscale captures supportive experiences. The Spanish version shows strong internal consistency α = 0.92.

#### 2.2.7 The Inventory of Complicated Grief‐Revised (ICG‐R)

The ICG‐R (Prigerson et al. 1995; Limonero et al. 2009) consists of 19 items assessing grief‐related thoughts and behaviours, rated on a five‐point Likert scale. Scores above 25 indicate complicated grief. The Spanish version showed a good internal consistency α = 0.88.

### 2.3 Procedure

Data were collected via an online survey on Lime Survey. Recruitment occurred through two main channels: suicide survivor associations in Spain, which shared the survey with members, and public calls on social media in collaboration with influential suicide loss accounts, like @stop.suicidios. Dissemination was free and uncompensated. Participation was voluntary, anonymous and required informed consent.

### 2.4 Statistical analysis

The data were analysed using IBM SPSS Statistics (Version 27) for Windows (IBM Corp 2020). Descriptive statistics were used to summarize key variables, while Pearson's correlations examined relationships between them. Multiple regression analyses were conducted to assess the predictive strength of various factors. Additionally, a one‐way analysis of variance (ANOVA) was performed to evaluate differences in prolonged grief symptoms, depressive symptoms and posttraumatic growth based on the participant's relationship to the deceased.

## 3 Results

Table 2 presents a summary of descriptive statistics for the different variables measured among the participants.

**TABLE 2:** Descriptive statistics.

	Mean	*SD*	Min	Max	Shapiro–Wilk	
					W	*p*
Time since loss (years)	6.05	8.30	0.08	40		
Age of the deceased (years)	42.10	18.87	18	94		
Symptoms of Prolonged Grief	35,5	15,6	3	71	0,98	< 0.001
Posttraumatic Growth	52,5	24,3	0	102	0,98	0.007
Depressive symptoms	11,1	7,9	0	27	0,93	< 0.001
Social support	15,4	4,9	5	25	0,97	< 0.001
Meaning in Life	43,2	16,1	10	70	0,95	< 0.001
SMILES‐invalidation	46,6	11,7	15	73	0,98	0.012
SMILES‐validation	31,3	6,2	11	45	0,98	< 0.001

*Note:* SD. standard deviation; Min. minimum value; Max. maximum value; W. the test statistic; *p*. significance values.

### 3.1 Correlations Analysis

Table 3 presents the Pearson correlation coefficients among the main study variables. Prolonged grief symptoms showed significant positive correlations with depressive symptoms (*r* = 0.640, *p* < 0.01) and social invalidation (*r* = 0.660, *p* < 0.001), and significant negative correlations with meaning in life (*r* = −0.747, *p* < 0.01), social support (*r* = −0.240, *p* < 0.001) and social validation (*r* = −0.343, *p* < 0.001). Moreover, post‐traumatic growth showed significant positive correlations with meaning in life (*r* = 0.430, *p* < 0.01) and social validation (*r* = 0.495, *p* < 0.001), and significant negative correlations with social invalidation (r = −0.156, *p* < 0.01) and depressive symptoms (*r* = −0.137, *p* < 0.05). Additionally, depressive symptoms were significantly positively correlated with social invalidation (*r* = 0.426, *p* < 0.001), and significantly negatively correlated with meaning in life (*r* = −0.747, *p* < 0.001), social validation (*r* = −0.266, *p* < 0.001) and social support (*r* = −0.130, *p* < 0.05).

**TABLE 3:** Correlation analysis of key variables: time elapsed, symptoms of prolonged grief, posttraumatic growth, depressive symptoms, social support, meaning in life, SMILES‐invalidation, and SMILES‐validation.

		1	2	3	4	5	6	7
1	Time elapsed since death	—						
2	Symptoms of Prolonged Grief	−0.192**	—					
3	Posttraumatic Growth	0.028	−0.136*	—				
4	Depressive symptoms	−0.107	0.640***	−0.137*	—			
5	Social support	−0.006	−0.240***	0.049	−0.130*	—		
6	Meaning in Life	0.098	−0.599***	0.430***	−0.747***	0.187**	—	
7	SMILES‐invalidation	−0.120*	0.660***	−0.156**	0.426***	−0.225***	−0.429***	—
8	SMILES‐validation	0.021	−0.343***	0.495***	−0.266***	0.104	0.406***	−0.299***

*** Significant correlation at 0.001 level.

** Significant correlation at 0.01 level.

* Significant correlation at 0.05 level.

### 3.2 Regression Analysis

#### 3.2.1 Prediction of Prolonged Grief Symptoms

The first regression analysis aimed to predict the score on prolonged grief symptoms. The analysis was conducted in two steps (Table 4).

**TABLE 4:** Regression analysis predicting symptoms of prolonged grief, posttraumatic growth, and depressive symptoms based on time elapsed, age of deceased, social support, meaning in life, SMILES‐invalidation, and SMILES‐validation.

Predictor variables	Symptoms of prolonged grief					Posttraumatic growth					Depressive symptoms				
	*B*	*SE*	*β*	*t*	*p*	*B*	*SE*	*β*	*t*	*p*	*B*	*SE*	*β*	*t*	*p*
First step															
Age of deceased	−0.136	0.049	−0.167	−2.76	0.006	−0.102	0.080	−0.080	−1.27	0.202	−0.067	0.026	−0.160	−2.61	0.009
Time elapsed since death	−0.227	0.073	−0.188	−3.11	0.002	0.073	0.118	0.039	0.62	0.536	−0.058	0.038	−0.093	−1.51	0.131
Second step															
Age of deceased	−0.039	0.035	−0.047	−1.11	0.265	−0.135	0.067	−0.105	−1.99	0.047	−0.016	0.018	−0.038	−0.89	0.369
Time elapsed since death	−0.123	0.050	−0.102	−2.44	0.015	0.035	0.098	0.018	0.35	0.723	−0.010	0.026	−0.017	−0.40	0.689
Social support	−0.187	0.135	−0.059	−1.38	0.168	−0.027	0.263	−0.005	−0.10	0.919	0.059	0.068	0.036	0.86	0.390
Meaning in life	−0.363	0.046	−0.378	−7.86	0.000	0.517	0.090	0.343	5.75	0.000	−0.352	0.023	−0.714	−15.05	0.000
SMILES‐invalidation	0.634	0.066	0.472	9.63	0.000	0.338	0.128	0.161	2.64	0.009	0.110	0.033	0.160	3.31	0.001
SMILES‐validation	0.068	0.136	0.024	0.49	0.619	1.895	0.265	0.424	7.15	0.000	0.132	0.069	0.090	1.90	0.057

*Note:* B. B coefficients; SE. standard errors; β. standardized beta coefficients; *t*. *t*‐values; *p*. significance values.

In the first step, the model explained 6.8% of the variance (*R* ^*2*^ = 0.068, *R* ^*2*^_adjusted_ = 0.061, *F*_(2, 258)_ = 9.47, *p* = 0.000). In the second step, the inclusion of psychological and social variables significantly increased the explained variance to 56% (*R* ^*2*^ = 0.571, *R* ^*2*^_adjusted_ = 0.561, *F*_(6, 254)_ = 56.40, *p* = 0.000), suggesting that these factors have a substantial impact on the dependent variable. The regression coefficients (Table 3) revealed that the deceased's age (*β* = −0.136, *p* = 0.006) and time elapsed since the death (*β* = −0.227, *p* = 0.002) were significant predictors in the first step. In the second step, meaning in life (*β* = −0.363, *p* = 0.000) and social invalidation (*β* = 0.634, *p* = 0.000) showed significant associations with the dependent variable. In contrast, social validation and perceived social support were not a significant predictor.

#### 3.2.2 Prediction of Depressive Symptomatology

A second regression analysis was conducted to predict depressive symptoms. The analysis was conducted in two steps (Table 4).

In the first step, the model explained 2.9% of the variance (*R* ^*2*^ = 0.037, *R* ^*2*^_*adjusted*_ = 0.029, *F*_(2, 258)_ = 4.95, *p* = 0.008). In the second step, the inclusion of psychological and social variables significantly improved the explained 57.1% of the variance (*R* ^*2*^ = 0.581, *R* ^*2*^_*adjusted*_ = 0.571, *F*_(6, 254)_ = 58.76, *p* = 0.000), indicating that these factors have a considerable impact on depressive symptoms. Regression coefficients (Table 3) showed that, in the first step, the deceased's age (*β* = −0.067, *p* = 0.009) was a significant predictor. In the second step, meaning in life (*β* = −0.352, *p* = 0.000) and social invalidation (*β* = 0.110, *p* = 0.001) were significantly associated with depressive symptoms. However, social validation, perceived social support and time elapsed since the loss were not significant predictors.

#### 3.2.3 Prediction of Posttraumatic Growth

A third regression analysis was performed to predict posttraumatic growth. The analysis was conducted in two steps (Table 4).

In the first step, the model was not significant and did not explain the variance (*R* ^*2*^ = 0.007, *R* ^*2*^_*adjusted*_ = 0.000, *F*_(2, 258)_ = 0.948, *p* = 0.389). However, in the second step, the inclusion of psychological and social variables significantly improved the explained variance by 32.5% (*R* ^*2*^ = 0.340, *R* ^*2*^_*adjusted*_ = 0.325, *F*_(6, 254)_ = 21.82, *p* = 0.000), indicating that these factors play an essential role in the dependent variable. Regression coefficients (Table 4) revealed that, in the second step, the deceased's age (*β* = −0.067, *p* = 0.009), meaning in life (*β* = −0.352, *p* = 0.000), social invalidation (*β* = 0.110, *p* = 0.001) and social validation (*β* = 0.110, *p* = 0.001) were significant predictors of the dependent variable. In contrast, perceived social support and time elapsed since the death were not a significant predictors.

### 3.3 Analysis of Variance (ANOVA) based on Relationship to the Deceased

A one‐way analysis of variance (ANOVA) was conducted to assess differences in prolonged grief symptoms, posttraumatic growth and depressive symptoms based on the participant's relationship to the deceased. Participants were categorized into six groups according to their relationship: parents (those who lost a child), children (those who lost a parent), close relatives (those who lost a sibling or grandchild), extended family (those who lost an uncle, nephew, cousin, or brother/sister‐in‐law), friends and partners (those who lost a spouse or romantic partner).

The ANOVA results indicated statistically significant differences in prolonged grief symptoms (*F*_(5,255)_ = 4.998, *p* = 0.000) and in depressive symptoms (*F*_(5,255)_ = 2.533, *p* = 0.029), while no statistically significant differences were found in posttraumatic growth (*F*_(5,255)_ = 0.879, *p* = 0.496). To determine which specific groups differed from each other, multiple comparisons were performed using Bonferroni's post hoc test, with an adjusted significance level of α = 0.05.

In the analysis of prolonged grief symptoms, results showed that participants who had lost a child reported the highest scores (*M* = 41.32, *SD* = 14.62), followed by those who had lost a partner (*M* = 36.70, *SD* = 15.50) or a parent (*M* = 36.24, *SD* = 15.39). At an intermediate level were those who had lost a close relative (sibling or grandchild) (*M* = 33.64, *SD* = 15.39) and those who had lost a friend (*M* = 32.92, *SD* = 15.15). The lowest levels of prolonged grief symptoms were found in those who had lost an extended family member (uncle, nephew, cousin or brother/sister‐in‐law) (*M* = 25.19, *SD* = 15.78). Bonferroni's test revealed that losing a child resulted in significantly higher levels of prolonged grief symptoms compared to losing an extended family member (*M*_*diff*_ = 16.13, *SE* = 3.62, *p*_*bonferroni*_ = 0.000), a friend (*M*_*diff*_ = 8.39, *SE* = 2.61, *p*_*bonferroni*_ = 0.022) and a close relative (*M*_*diff*_ = 7.67, *SE* = 2.55, *p*_*bonferroni*_ = 0.044).

Regarding depressive symptoms, participants who had lost a partner showed the highest scores (*M* = 14.35, *SD* = 6.79), followed by those who had lost a child (*M* = 12.59, *SD* = 8.23). Lower levels of depressive symptoms were observed in participants who had lost a parent (*M* = 10.56, *SD* = 8.50), a friend (*M* = 10.92, *SD* = 7.32) or a close relative (*M* = 9.32, *SD* = 7.99), while the lowest scores were found in the extended family group (*M* = 8.09, *SD* = 7.08). Bonferroni's test did not show statistically significant differences between the groups (*p* > 0.05).

Finally, in relation to post‐traumatic growth, the results did not show statistically significant differences between the groups (*p* = 0.496), with average scores ranging between *M* = 49.10 and *M* = 57.83.

## 4 Discussion

This study examined relationships between prolonged grief symptoms, depressive symptoms, post‐traumatic growth, meaning in life, social support, social validation/invalidation and time since death in Spanish adults bereaved by suicide. Greater meaning in life, social support and validation were associated with lower prolonged grief symptoms and depressive symptoms, highlighting their protective role in adjustment. These findings align with research by Oexle and Sheehan (2020), who emphasized the benefits of perceived social support for emotional well‐being. The study also identified key demographic, circumstantial and psychosocial predictors of prolonged grief symptoms, depressive symptoms and post‐traumatic growth.

First, results indicated that social invalidation was the strongest predictor of prolonged grief symptoms, demonstrating that experiences of stigma and lack of understanding from one's social environment may exacerbate grief‐related symptomatology. This finding is consistent with prior research that has highlighted the negative impact of stigma and lack of support on grief adaptation following a suicide (Hanschmidt et al. 2016; Levi‐Belz and Lev‐Ari 2019; Pitman et al. 2018). Furthermore, perceived social invalidation can lead to feelings of isolation and hopelessness, hindering coping and readjustment (Cerel et al. 2019).

In contrast, a greater level of meaning in life was associated with lower levels of prolonged grief symptoms, in line with studies emphasizing the importance of meaning reconstruction after a loss (Neimeyer 2006). This process not only helps mitigate emotional pain but also fosters a narrative of resilience and recovery, as suggested by theoretical models of grief adaptation (Tedeschi and Calhoun 2004). Likewise, time elapsed since loss predicted lower levels of prolonged grief symptoms, supporting the notion that prolonged grief symptoms tend to gradually decline. Previous research has demonstrated that the first years after the loss are the most critical and that, over time, many survivors develop coping strategies that help them alleviate their pain (Brown et al. 2024; Oexle and Sheehan 2020; Whittaker et al. 2024). For example, Mitchell et al. (2004) noted that the early years following a loss tend to be the most challenging, with heightened levels of emotional distress.

Moreover, results indicated that social validation and invalidation were significant predictors of depressive symptoms. This reinforces the idea that feeling judged or misunderstood can have a detrimental impact on the mental health of suicide loss survivors (Pitman et al. 2018). Additionally, a greater sense of meaning in life was found to predict lower levels of depressive symptoms, indicating that finding meaning in life as a way to reconstruct the loss may serve as a protective factor against depressive symptoms.

These findings highlight that for survivors of suicide loss, feeling misunderstood or judged by their social environment can significantly worsen their grief process, intensify depressive symptoms and prolong emotional suffering. This underscores the need for therapeutic interventions to not only address depressive symptoms but also work towards reducing perceptions of social invalidation to facilitate better adaptation following loss (Bellet et al. 2018; Marek and Oexle 2024). In this regard, Froese et al. (2020) emphasize that leisure activities and social interactions can play a crucial role in survivors' recovery by providing spaces for emotional relief and moments of respite. They also highlight the importance of constructing meaning from the loss, as this allows survivors to rebuild their sense of purpose in life, which may be key to overcoming pain and despair.

Unlike prolonged grief symptoms and depressive symptoms, post‐traumatic growth was more closely linked to social validation, indicating that feeling understood and supported in reconstructing one's personal narrative facilitates positive transformation following loss (Tedeschi and Calhoun 2004). This finding is consistent with studies showing that positive social interactions can foster trauma reinterpretation and facilitate psychological adaptation (Neimeyer et al. 2023). However, concerning post‐traumatic growth, results did not reveal significant differences based on time elapsed since the loss. These findings are in line with Whittaker et al. (2024), who found that while prolonged grief symptoms and depressive symptoms decrease over time, post‐traumatic growth does not solely depend on the passage of time. Instead, factors such as social support and social validation are critical in helping survivors find new purpose and experience personal growth. This process does not occur automatically but is often the result of prolonged and deliberate efforts to reevaluate and rebuild life after the loss (Hurst and Kannangara 2024). Social validation not only alleviates emotional suffering but also facilitates the construction of a renewed life purpose, which is essential for positive post‐traumatic growth (Quezada‐Berumen and González‐Ramírez 2020).

Research suggests that prolonged grief symptoms and post‐traumatic growth reflect distinct psychological processes. Prolonged grief symptoms are linked to fixation on the past and persistent rumination, leading to unresolved pain (Neimeyer 2006), while post‐traumatic growth involves a future‐oriented shift, positive reinterpretation and personal growth (Tedeschi and Calhoun 2004). This temporal contrast may explain why they do not always coexist.

Although the deceased's age initially seemed to affect prolonged grief symptoms, this effect disappeared when controlling for variables like meaning in life and social invalidation, indicating it was not a significant predictor. This supports findings that age is not consistently linked to grief intensity, whereas kinship, such as losing a child or spouse, is more influential (Parro‐Jiménez et al. 2021).

Another objective was to examine the impact of kinship on prolonged grief symptomatology. Results showed significant differences: parents who lost a child reported the highest levels of prolonged grief symptoms, supporting research that identifies child loss as particularly devastating (Szuhany et al. 2021). In contrast, those who lost a distant relative or friend had significantly lower prolonged grief symptoms, highlighting the role of kinship in grief intensity (Brown et al. 2024).

Similar patterns were found for depressive symptoms, with higher depressive symptoms among those who lost a partner or child, reinforcing the emotional impact of close relationships (Parro‐Jiménez et al. 2021). No significant differences in post‐traumatic growth were found by relationship, suggesting that post‐traumatic growth may rely more on individual and social factors than on kinship.

In conclusion, our findings identify social invalidation as a key risk factor for both prolonged grief symptoms and depressive symptoms, while meaning in life consistently acts as a protective factor across models. Post‐traumatic growth, however, was more strongly associated with social validation than with invalidation. Grief impact also varied by relationship, with parents and partners showing the highest levels of prolonged grief symptoms and depressive symptoms. These results underscore the need for interventions that reduce social invalidation and support meaning reconstruction to foster healthier adaptation to suicide‐related grief.

## 5 Strengths, Limitations and Future Directions

This study on prolonged grief after suicide is notable for its specific sample of Spanish suicide bereaved individuals. Data analysis reveals temporal variations in grief. However, its cross‐sectional design and recruitment via networks limit generalizability and preclude causal inference. Additionally, no officially validated Spanish version of the Inventory of Social Support (ISS) was available at the time of the study, which may affect the interpretability of that measure. Future research should employ longitudinal designs, broader samples and focus on interventions promoting social support and meaning‐making.

## Data Availability

The data that support the findings of this study are available on request from the corresponding author. The data are not publicly available due to privacy or ethical restrictions.
